# Tests of association based on genomic windows can lead to spurious associations when using genotype panels with heterogeneous SNP densities

**DOI:** 10.1186/s12711-021-00638-x

**Published:** 2021-05-26

**Authors:** Jinghui Li, Zigui Wang, Rohan Fernando, Hao Cheng

**Affiliations:** 1 Department of Animal Science, University of California, Davis, USA; 2 Department of Animal Science, Iowa State Univeristy, Ames, USA

## Abstract

Dense single nucleotide polymorphism (SNP) panels are widely used for genome-wide association studies (GWAS). In these panels, SNPs within a genomic segment tend to be highly correlated. Thus, association studies based on testing the significance of single SNPs are not very effective, and genomic-window based tests have been proposed to address this problem. However, when the SNP density on the genotype panel is not homogeneous, genomic-window based tests can lead to the detection of spurious associations by declaring effects of genomic windows that explain a large proportion of genetic variance as significant. We propose two methods to solve this problem.

## Background

Genome-wide association studies (GWAS) have been widely used to locate quantitative trait loci (QTL) for traits of interests [1–3]. Typically, GWAS methods are based on testing the significance of single nucleotide polymorphism (SNP) effects. However, SNPs within a genomic segment can be highly correlated with each other and jointly influence the phenotype, which makes the effect of a single SNP difficult to be identified [4]. Therefore, GWAS based on testing genomic windows have been proposed to overcome this problem [5].

Many approaches have been developed to make inferences of associations based on genomic windows. Chen et al. [6] generalized a frequentist method to test genomic window effects through extending the popular single SNP testing method, Efficient Mixed-model Association eXpedited (EMMAX) [7]. It has been suggested that EMMAX is not statistically coherent since it treats one marker (or window) effect as both fixed and random, and repeats the process for every single marker (or window) to make inferences [6, 8]. Other frequentist methods make inferences on genomic windows using bootstrap or sample permutation [4, 9], which are computationally expensive. Therefore, the use of Bayesian approaches, which include all marker effects simultaneously, have gained popularity in recent research. Legarra et al. [10] developed a method of Bayes factors to evaluate genomic windows, but did not sufficiently justify the threshold that was used. Fernando et al. [11] described a method to calculate the window posterior probability of association (WPPA) using Markov chain Monte Carlo (MCMC). They showed that the posterior type I error rate is less than $\alpha$ when declaring an association for a genomic window with WPPA $>1 - \alpha$. However, we will show that this approach may result in spurious associations, i.e., the proportion of false positives exceeding the expectation, for genomic windows at the higher end of the distribution for SNP density. Note that some frequentist methods, e.g., [12], may also detect spurious associations if effects of genomic windows explaining a large proportion of genetic variance are declared as significant.

The objectives of this study are to show that genomic-window based tests can lead to the detection of spurious associations when the SNP density on the genotype panel is not homogeneous and to propose two methods to solve this problem. The article is composed of three parts. First, we will demonstrate the problem of spurious associations in analyzing a real data set. Second, two methods to overcome this problem are described and compared with the original method. Finally, the validity of our methods is examined through simulated data sets.

## Methods

### Demonstration of spurious associations

#### Bayesian regression models

For simplicity of the description, all analyses in this study were based on a single-trait regression model with a general mean as the only fixed effect. The model for individual *i* from *n* genotyped individuals can be written as:

$$\begin{aligned} y_i = \mu + \sum _{j=1}^p m_{ij} \alpha _j + e_i, \end{aligned}$$

where $y_i$ is the phenotypic value for individual *i*, $\mu$ is the overall mean, $m_{ij}$ is the genotype covariate at locus *j* for individual *i* (coded as 0, 1 or 2), $\alpha _j$ is the marker effect for locus *j*, and $e_i$ is the random residual for individual *i*. We assume that $\mu$ has a flat prior, and all the residuals, $e_i$, are independent and identically distributed normal variables with null mean and variance $\sigma _e^2$, which in turn is assumed to have a prior of a scaled inverse chi-square distribution. The prior used in BayesC$\pi$ [13], a Bayesian variable selection method, is used for the marker effects. In BayesC$\pi$, the prior for marker effects, $\alpha _j$, is identical and independent with mixture distributions, each of which has a point mass at zero with probability $\pi$, and a normal distribution with probability $1 - \pi$ having a null mean and variance $\sigma _\alpha ^2$, which in turn has a prior of a scaled inverse chi-square distribution. In addition, $\pi$ is treated as unknown with a uniform prior.

#### Inference of associations based on genomic windows

Inferences of associations based on genomic windows were made using the method described by Fernando et al. [11]. The posterior distribution for the proportion of the genetic variance explained by each genomic window is estimated from MCMC samples as follows. First, the genotypic value of the genomic window *w* is calculated as:

$$\begin{aligned} \mathbf {g}_w = \mathbf {M}_w \varvec{\alpha }_w, \end{aligned}$$

where $\mathbf {M}_w$ is the genotype covariates matrix and $\varvec{\alpha }_w$ is the vector of the samples of marker effects for SNPs in the window *w*. Then, the genetic variance explained by window *w* is calculated as:

$$\begin{aligned} \sigma _{g_w}^2 = \frac{\sum _{i=1}^{n} g_{w_i}^2}{n} - \left(\frac{\sum _{i=1}^{n} g_{w_i}}{n}\right)^2, \end{aligned}$$

where $g_{w_i}$ is the local genotypic value of window *w* for individual *i* and *n* is the total number of individuals. Similarly, the total genetic variance, $\sigma _g^2$, is calculated as:

$$\begin{aligned} \mathbf {g}= & {} \mathbf {M} \varvec{\alpha }\\ \sigma _g^2= & {} \frac{\sum _{i=1}^{n} g_i^2}{n} - \left(\frac{\sum _{i=1}^{n} g_i}{n}\right)^2, \end{aligned}$$

where $\mathbf {g}$ is the vector of genotypic values, $\mathbf {M}$ is the genotype covariate matrix, $\varvec{\alpha }$ is the vector of the samples of marker effects and $g_i$ is the genotypic value for individual *i*. Finally, the proportion of the genetic variance explained by genomic window *w* is:

$$\begin{aligned} q_w = \frac{\sigma _{g_w}^2}{\sigma _g^2}, \end{aligned}$$

Given the MCMC samples of $q_w$, WPPA is calculated as the proportion of $q_w$ samples that exceed the value of *T*. A constant value of $T=0.1\%$ was used for all windows in [11], and others have used $T=\frac{1}{N}$, where *N* is the total number of genomic windows [14].

#### Real data

An *Oryza sativa* data set [15] with 413 *Oryza sativa* individual records of genotypes and the trait of flowering time in Arkansas was used to demonstrate the problem of spurious associations. After removing the records with missing data for the trait and genotypes with a minor allele frequency less than 0.05, 374 individuals and 33,701 SNPs were included in the analysis. The data are publicly available at the Rice Diversity Panel (http://www.ricediversity.org). Inferences of association based on genomic windows using Bayesian regression models were conducted using non-overlapping windows of size 1 Mb. The genome of the real data was divided into 378 non-overlapping genomic windows of size of 1 Mb, and the value of *T* was set to $\frac{1}{N}$, where *N* = 378. The lower, median and upper quartiles for the number of SNPs per window ($p_w$) were 60, 82 and 106, respectively. Seven windows containing more than 175 (i.e., upper quartile + $1.5 \times$ (upper quartile − lower quartile)) SNPs were identified as dense.

### Two solutions

The results of the analysis with real data showed false positives on dense genomic windows, which will be demonstrated in the "Results and discussion" section. Here, we propose two methods to solve the problem of false positives. We denote the above method of making inferences of associations based on genomic windows as 1 MbT. The first method is to use a window size of 100 SNPs (100 T) and set the value *T* to $\frac{1}{N'}$, where $N'$ is the total number of windows for a window size of 100 SNPs. This method (100 T) provides equal numbers of SNPs for each window, and thus the problem caused by window density can be avoided. However, a window size of 100 SNPs may not be as informative as a window size of 1 Mb. Therefore, using window-specific *T* for a window size of 1 Mb (1 MbTw) is proposed as the second method. Here, instead of a constant *T*, a set of window-specific $T_w = \frac{p_w}{p}$ is used, where $p_w$ is the number of SNPs in window *w* and *p* is the total number of SNPs.

### Data analysis using two solutions

The Rice Diversity Panel data were reanalyzed using the two methods proposed above. In order to examine the properties of the solutions, the three methods, 1 MbT, 100 T, and 1 MbTw, were also compared through a simulation study. Phenotypes were simulated based on the genotypic data from the Rice Diversity Panel. Since the number of QTL ($n_{QTL}$) may influence the GWAS result, 90 phenotypic data sets were simulated for $n_{QTL} =$ 30, 90, and 300 (30 replicates for each). All the QTL effects were generated from a standard normal distribution. In order to show the false positive caused by dense windows, all the QTL positions were randomly selected from the SNPs located on nondense windows (i.e., all windows except the seven dense ones identified previously). Phenotypes were generated based on a heritability of 0.5.

The GWAS performance of the three methods was compared using the area under receiver operating characteristic (AUC), which was calculated through the R package ROCR [16]. In order to exclude the irrelevant AUC with low levels of specificity, only the partial area under the curve up to the false positive rate of 5% (pAUC5) was calculated [6]. For ease of comparison, all pAUC5 values were rescaled such that the pAUC5 of a random classifier equals 1 (i.e., all pAUC5 values were divided by 0.00125). The pAUC5 values of 1 MbT, 1 MbTw and 100 T were compared for each level of $n_{QTL}$ by ANOVA F-test at a significance level of 0.05.

We also conducted the GWAS based on EMMAX using a window size of 1 Mb [6] denoted as 1 MbFre, in order to compare our methods with a frequentist method. The significance of each window was inferred through a $\chi ^2$ test and the result is shown as the negative logarithm of p-values.

All analyses based on Bayesian regression methods were performed using the JWAS package [17], which is an open-source, publicly available package for single-trait and multi-trait whole-genome analyses. The GWAS based on EMMAX was performed in R [18].

## Results and discussion

### Real data analysis

In the GWAS result based on 1 MbT, the correlations of $p_w$ with $q_w$ and WPPA were 0.62 and 0.65, respectively, which means that dense windows tend to explain more genetic variance and have large WPPA. One possible explanation (hypothesis) for this observation is that a window with a high density of SNPs is also likely to contain more trait loci than a window with a lower density of SNPs. To test this hypothesis, we reanalyzed the data and computed the correlations of $p_w$ with $q_w$ and WPPA after phenotype permutation, in which we shuffled the phenotype labels but kept the genotypes the same. If this hypothesis was true, the correlations following permutation of the phenotypes should be low since the phenotype–genotype relationship is removed. However, in 30 replicates of random shuffling, the average correlation between $p_w$ and $q_w$ was 0.36, and that between $p_w$ and WPPA was 0.62. In addition, the largest WPPA was always obtained for one of the three densest windows, containing 733, 594 and 545 SNPs, respectively. The high correlation between $p_w$ and WPPA indicates that dense windows tend to have large WPPA regardless of the phenotypes and this is very likely to cause an excess of false positives. Use of priors in other approaches, such as BayesB [19, 20] or Bayesian LASSO [21], would result in the same problem as above.

**Fig. 1:**
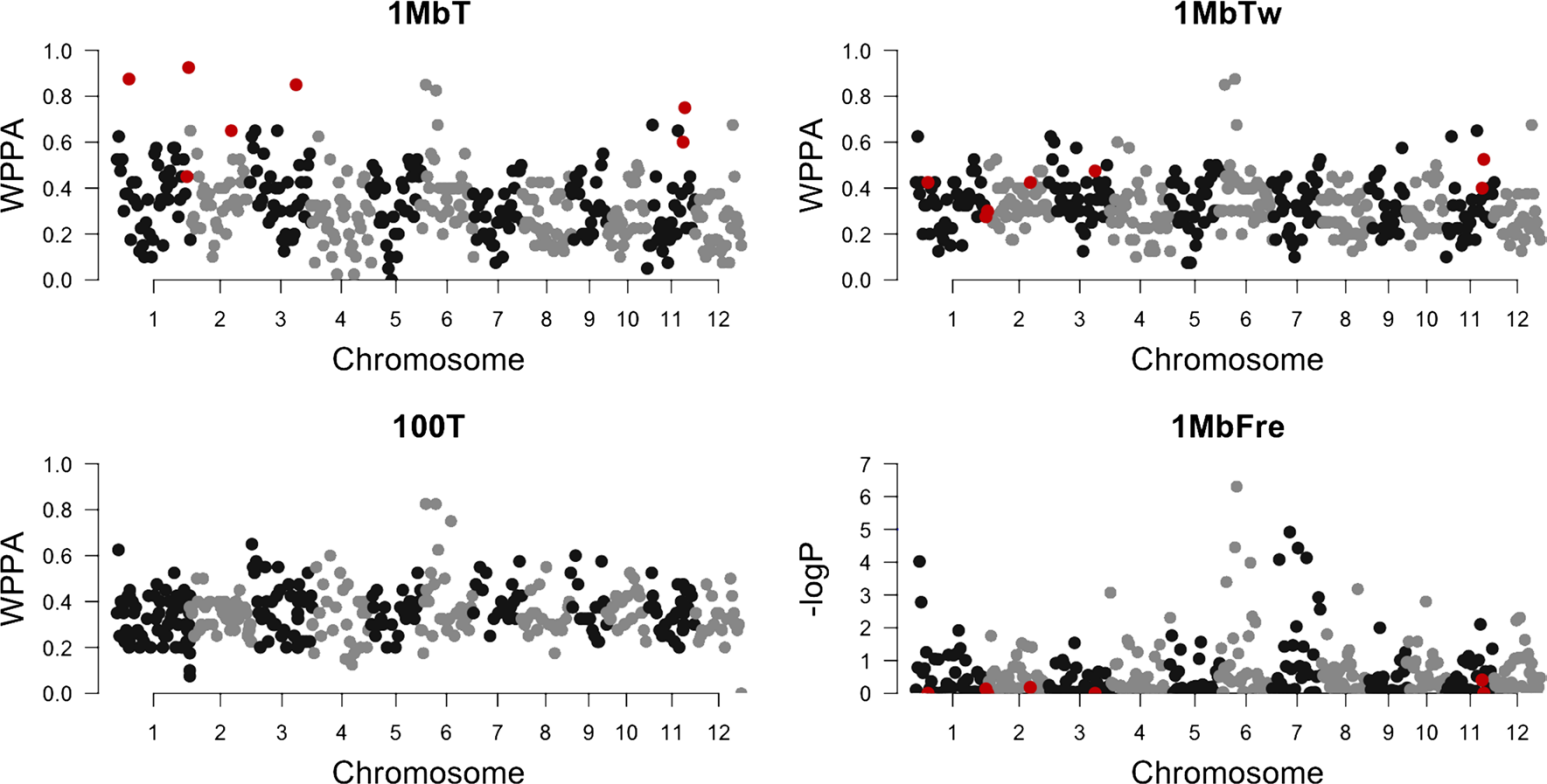
Manhattan plot of GWAS analysis for the real data. 1 MbT = GWAS analysis with a window size of 1 Mb and a constant *T* for all windows; 1 MbTw = GWAS analysis with a window size of 1 Mb and window specific *T*; 100 T = GWAS analysis with window size of 100 SNPs and a constant *T*; 1 MbFre = GWAS analysis with a window size of 1 MB using EMMAX. Red points represent dense windows

After changing the value of *T* from a constant to a window-specific value (1 MbTw), the correlation between $p_w$ and $q_w$ stayed the same, but that between $p_w$ and WPPA decreased from 0.65 to 0.29. The Manhattan plot shows that the dense windows showing significant signals disappeared when using 1 MbTw instead of 1 MbT (Fig. 1). One should note that, the use of a window-specific *T* does not change the proportion of genetic variance explained by each window, but it changes the WPPA through different values for *T*.

### Simulated data analysis

**Fig. 2:**
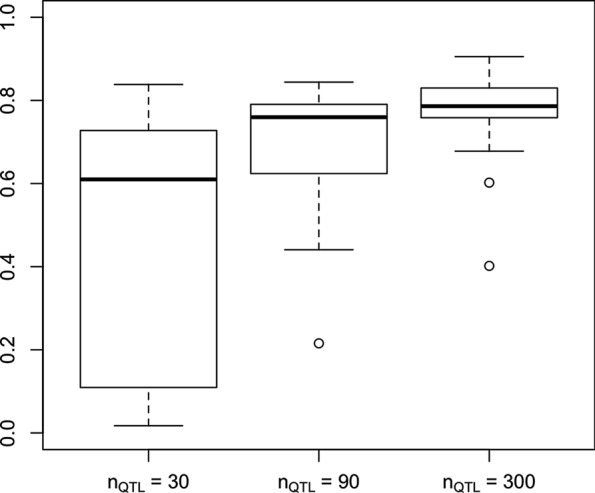
Correlations between number of SNPs and the proportion of genetic variance explained by each window with a window size of 1 Mb for simulated data. Correlations were obtained based on 30 replicates for each number of simulated QTL

**Fig. 3:**
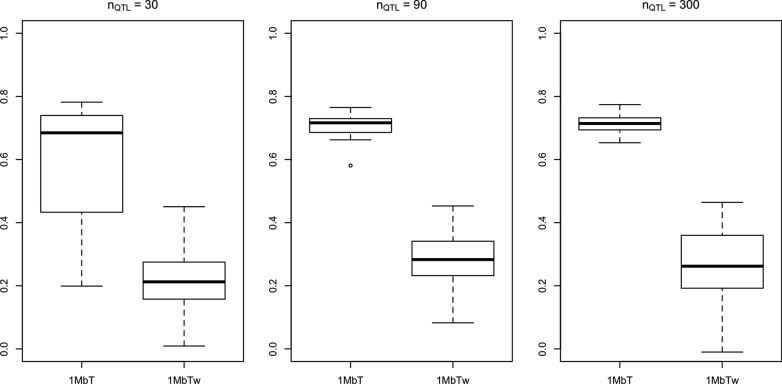
Correlations between number of SNPs and WPPA for each genomic window. Correlations were obtained based on 30 replicates for each number of simulated QTL. 1 MbT = GWAS analysis with a window size of 1 Mb and a constant *T* for all windows; 1 MbTw = GWAS analysis with a window size of 1 Mb and a window specific *T*

The correlations obtained with the simulation data are similar to those with real data. For a window size of 1 Mb, the correlation between $p_w$ and $q_w$ was strong (Fig. 2), especially for $n_{QTL} =$ 90 and 300. Window density was also highly correlated with WPPA when using a constant *T* value to calculate WPPA, while the correlation significantly decreased when using a window-specific *T* value (Fig. 3). When *T* is set to $\frac{1}{N}$, the null hypothesis is that each genomic window explains an equal amount of genetic variance (i.e., no QTL exists). However, when using $T_w = \frac{p_w}{p}$, the null hypothesis is that each SNP explains an equal amount of genetic variance, thus dense windows have to explain more genetic variance than non-dense windows to obtain the same WPPA value.

The pAUC5 values of 1 MbT, 1 MbTw and 100 T are shown in Table 1. Although no significant difference was found for pAUC5 when $n_{QTL} = 30$, pAUC5 of 1 MbT was significantly lower than that of 1 MbTw and 100 T when $n_{QTL}$ = 90 and 300. No significant difference was found between 1 MbTw and 100 T. The result of pAUC5 indicates that using a window size of 100 SNPs or a window-specific *T* improves the GWAS performance. Note that the performance of 100 T may not be comparable to that of 1 MbT and 1 MbTw because different genomic windows are tested in 100 T. Similar results were observed for simulation studies with different heritabilities (results not shown). The correlation between the negative logarithm of p-values and the number of SNPs in each window was low (− 0.11) for 1 MbFre in the real data analysis, and none of the dense windows was significant (Fig. 1). This is because, under the null hypothesis, for each window, the test statistic follows a Chi-square distribution with the degrees of freedom equal to the number of SNPs in the window, which lowers the negative logarithm of p-values for dense windows. Regarding the GWAS performance in the simulation study, 1 MbFre performed significantly worse than 1 MbT and 1 MbTw when $n_{QTL}$ = 30 and 90 based on pAUC5, although no significant difference was observed when $n_{QTL}$ = 300. We also conducted another simulation study using a swine dataset composed of 928 individuals and 44,055 SNPs [22]. In the swine dataset, the 1 MbTw consistently improved the GWAS performance compared to 1 MbT when using genotype panels with heterogeneous SNP densities (results not shown).

**Table 1:** Average rescaled partial areas under a receiver operating characteristic curve up to a false positive rate of 5% (pAUC05) for 1 MbT, 1 MbTw and 100 T

	1 MbT	1 MbTw	100 T	1 MbFre
$n_{QTL} = 30$	$4.38^a$	$5.27^a$	$5.29^a$	$2.37^b$
$n_{QTL} = 90$	$1.49^b$	$2.68^a$	$2.19^a$	$1.03^b$
$n_{QTL} = 300$	$0.52^b$	$1.58^a$	$1.48^a$	$1.30^a$

Means were obtained based on 30 replicates for each number of simulated QTL. 1 MbT = GWAS analysis with a window size of 1 Mb and a constant *T* for all windows; 1 MbTw = GWAS analysis with a window size of 1 Mb and window specific *T*; 100 T = GWAS analysis with a window size of 100 SNPs and a constant *T*; 1 MbFre = GWAS analysis with a window size of 1 MB using EMMAX. Different superscripts in the same row indicate a significant difference ($p < 0.05$)

## Conclusion

When SNP densities on the genotype panel are heterogeneous, genomic-window based tests can lead to the detection of spurious associations by declaring effects of genomic windows that explain a large proportion of genetic variance as significant. Two straightforward solutions are offered in this paper. One is to make each window contain an equal numbers of SNPs, and the other one is to use a window-specific *T* value, so that false positives caused by window density may be avoided.
